# Sepsis: personalization v protocolization?

**DOI:** 10.1186/s13054-019-2398-5

**Published:** 2019-06-14

**Authors:** Mervyn Singer

**Affiliations:** Bloomsbury Institute of Intensive Care Medicine, Division of Medicine, University College London, London, UK

**Keywords:** Sepsis, Protocolization, Personalization, Outcomes, Evidence-based medicine, Biomarkers, Diagnostics, Theranostics

## Abstract

The founding tenet of evidence-based medicine is to combine best evidence with clinical expertise. As David Sackett opined ‘Without clinical expertise, practice risks becoming tyrannised by evidence’. Rigid protocols and mandates, based on an inconclusive and low-level evidence base, cannot suit the physiological, biochemical and biological heterogeneity displayed by the individual septic patient. Indeed, clear proof of outcome benefit through adoption of an inflexible management approach is lacking and will certainly be detrimental to some. Therapy thus needs to be tailored to meet the individual patient’s needs. The same principle should be applied to clinical trials; the continued disappointments of multiple investigational strategies trialled over three decades, despite (often) a sound biological rationale, suggest a repeated methodological failure that does not account for the marked heterogeneity within the septic patient’s biological phenotype and thus marked variation in their host response. The increasing availability of rapid point-of-care diagnostics and theranostics should facilitate better patient selection and titrated optimization of the therapeutic intervention.

## Introduction

The history of intensive care has been littered with too many false dawns. Old management dogma, now derided, have been replaced by new and equally resolute convictions, many of which will, in time, undoubtedly follow a similar course. Retired bundles of care have been replaced by new bundles driven by clinical evangelists and government diktat. Yet these too lack a strong evidence base and can be readily challenged. Homogenizing treatment given to a highly heterogenous patient population that fall under the wide syndromic umbrella of sepsis may offer a minimum standard of care though at the potential cost of compromising outcomes in an individual patient. Likewise, the repeated multicentre trial failures of novel interventional strategies over many decades highlight the need to focus more precisely on the individual’s biological phenotype and adopt a personalized treatment approach. This article will briefly cover these issues and offer directions for the future.

## Guidelines or rules-of-stone?

Evidence-based medicine is intended to offer best treatment and represents a worthy aim to raise standards and homogenize care. It offers a framework when basic expertise is lacking. An excellent example is the Advanced Trauma Life Support course, established by an orthopaedic surgeon (James Styner) after a plane crash in rural Nebraska in 1976 on finding medical care for his injured children was disorganized and suboptimal. However, one size does not, and should not, fit all. While providing structure is clearly important, especially for less-experienced healthcare providers, it must be tailored to the individual patient. To cite David Sackett, one of the ‘godfathers’ of evidence-based medicine [1]:

*‘*Good doctors use both individual clinical expertise and the best available external evidence, and neither alone is enough. Without clinical expertise, practice risks becoming tyrannised by evidence, for even excellent external evidence may be inapplicable to or inappropriate for an individual patient.’

‘Evidence based medicine is not “cookbook” medicine. Because it requires a bottom up approach that integrates the best external evidence with individual clinical expertise and patients’ choice, it cannot result in slavish, cookbook approaches to individual patient care. External clinical evidence can inform, but can never replace, individual clinical expertise, and it is this expertise that decides whether the external evidence applies to the individual patient at all and, if so, how it should be integrated into a clinical decision. Similarly, any external guideline must be integrated with individual clinical expertise in deciding whether and how it matches the patient’s clinical state, predicament, and preferences, and thus whether it should be applied. Clinicians who fear top down cookbooks will find the advocates of evidence based medicine joining them at the barricades.’

Septic patients, especially when critically ill, represent a highly heterogenous population. It is over-simplistic to believe that the same strategy will benefit every patient as their underlying physiological reserve, underlying comorbidities, presenting physiology, unmeasured biological phenotype and response to drugs, fluids and other therapies will all vary markedly. Temporal variations during the acute illness must also be taken into consideration.

Sepsis management guidelines are worthy but, unfortunately, often taken too literally by clinical evangelists, hospitals, government bodies, lawyers and litigators with financial penalties, litigation and public ‘naming-and-shaming’ for non-compliant miscreants. Guidelines are thus forced into rigid protocols with little room for clinical judgement to be applied. We must acknowledge there is still no ‘right’ way to manage all the complexities of a septic patient. The overall evidence base for sepsis remains weak as shown by the limited number of ‘high-quality’ recommendations made by the Surviving Sepsis Campaign (SSC) [2]. Indeed, these recommendations mainly relate to interventions we should not do, rather than those we should. These guidelines are based on current consensus but, as the chequered history of sepsis management clearly shows, today’s strong belief is often tomorrow’s discard. The now-abandoned SSC 6-h and 24-h management bundles were heavily touted as ‘a group of interventions related to a disease process that, when executed together, result in better outcomes than when implemented individually. The individual bundle elements are built upon evidence-based practices. The science behind the elements of a bundle is so well-established that their implementation should be considered a generally accepted practice’ [3]. We were warned: ‘If all of the elements of the bundles are not incorporated .. your performance on the measures will suffer’ [3]. Unfortunately, this quasi-religious fervour has not been borne out by subsequent multicentre studies that failed to reproduce the original study findings, for example early goal-directed therapy [4, 5].

No one will argue that early identification and prompt, appropriate management are not important cornerstones of good treatment. However, what constitutes ‘appropriate’ is moot. For example, a fixed volume 30 ml/kg fluid resuscitation regimen within the current SSC bundle [2] will under-treat some yet over-treat others [6]. Patients cannot benefit from fluid overload [7], in much the same way that oxygen overload (hyperoxia)—an absolute iatrogenic phenomenon—is increasingly recognized as being harmful [8, 9].

A post hoc analysis [6] of the VASST trial, comparing vasopressin against norepinephrine in patients with septic shock, highlighted the increasing severity-adjusted mortality risk related to an increasing fluid balance, both at 12 h and 72 h after ICU admission (Table 1). Sicker patients will generally need more fluid than the less sick. However, while severity adjustment cannot account for all possible confounding factors, the disparity in fluid volumes given does strongly suggest a contribution to mortality from excess fluid. It is more rational to utilize appropriate monitoring to titrate fluid input to meet the needs of the patient in terms of adequacy of organ perfusion, rather than using an empiric, formulaic, unphysiologic approach.

**Table 1:** Relationship between fluid balance and mortality—retrospective analysis of the VASST study. Adapted from Boyd et al. [7]

	Quartile 1	Quartile 2	Quartile 3	Quartile 4
12-h fluid balance	710 (− 132–1480)	2880 (2510–3300)	4900 (4290–5530)	8150 (7110–10,100)
4-day fluid balance	1560 (− 723–3210)	8120 (6210–9090)	13,000 (11,800–14,700)	20,500 (17,700–24,500)
Adjusted hazard ratio vs quartile 4				
- 12 h	0.57 (0.41–0.80)	0.58 (0.41–0.82)	0.76 (0.56–1.03)	–
- 4 days	0.47 (0.30–0.72)	0.51(0.34–0.78)	0.74 (0.50–1.09)	–

Volumes expressed as median (inter-quartile range)

Likewise, antibiotics are thrown around indiscriminately and often to patients who are not septic and thus cannot gain from an inappropriate intervention. In different studies, only 57–80% of patients treated for suspected sepsis or admitted to intensive care units with septic shock were subsequently adjudicated as having ‘definite’ or ‘probable’ sepsis [10–12]. As discussed below, the evidence for benefit from early antibiotics in non-shock patients is questionable, and the propensity to harm significant yet under-recognized.

Despite this, a 2018 update from the SSC leadership argued that ‘the compelling nature of the evidence in the literature, which has demonstrated an association between compliance with bundles and improved survival in patients with sepsis and septic shock’ should mandate clinicians to combine the previous 3- and 6-h bundles into a single 1-h bundle, with time zero defined as the time of triage in the emergency department [13]. This 2018 guideline generated a fierce reaction particularly from the Emergency Medicine community, including an online campaign demanding retraction; at the time of writing, this petition had amassed over 6000 signatures [14].

## Do mandates work?

In the USA, Medicare/Medicaid has imposed ‘SEP-1’ (Sepsis National Hospital Inpatient Quality Measure) on emergency departments. This requires documentation of procedures that must be rigidly performed in patients with suspected sepsis, and detailed written justifications where any deviation occurs. Though currently used for reporting purposes, this is likely to be soon applied to hospital re-accreditation and reimbursement. A recent systematic review concluded that ‘no high- or moderate-level evidence shows that SEP-1 or its hemodynamic interventions improve survival in adults with sepsis’ [15]. The authors noted that SEP-1 requires reporting of up to five haemodynamic interventions, as many as 141 tasks, and as long as 3 h of documentation time for a single patient.

The National Health Service in England withholds 4% of each hospital’s budget with 1% being returned when targets are met for each of four annual CQUINs (quality improvement initiatives). A current CQUIN [16] requires use of early antibiotics within an hour of diagnosis of sepsis in emergency department patients. While an improvement over a previous mandate of ‘one hour from hospital arrival’, this has still driven a marked increase in use of antibiotics in the emergency department, in particular with agents such as meropenem and piptazobactam that, ironically, the CQUIN also seeks to restrict because of valid concerns over antimicrobial resistance. This strategy has failed to show any clear outcome benefit; in patients with a suspicion of sepsis admitted to English hospitals as emergencies, the number of hospital deaths, ICU bed days and 30-day readmission rates have not fallen [17]. Sadly, anecdotal evidence from some hospitals suggests antibiotics are being prescribed to meet these financial/quality improvement targets, even before the patient has been reviewed by a doctor.

From 2013, the New York State Department of Health required hospitals to follow ‘evidence-informed protocols’ based on the SEP-1 mandate for the early identification and treatment of sepsis. All hospitals were required to include a 3-h bundle with blood culture collection before administration of antibiotics, measurement of serum lactate and administration of broad-spectrum antibiotics, and a 6-h bundle requiring intravenous administration of 30 ml fluid/kg body weight in patients with hypotension or a serum lactate ≥ 4 mmol/l, initiation of vasopressors for refractory hypotension and re-measuring lactate within 6 h.

The headline finding of a retrospective analysis of 49,331 presumed septic patients in the New York State database [18] found patients had a higher risk-adjusted, in-hospital mortality (odds ratio 1.04 per hour; 95% CI, 1.03–1.06; *p* < 0.001) related to delayed administration of antibiotics. However, closer scrutiny of the presented data generates significant concerns as to the plausibility of this conclusion. The protocol was initiated within a 6-h window after arrival in the emergency department, rather than beginning at the time of entry. Actual in-hospital death was 22.6% in those completing the bundle within 3 h versus 23.6% in those completing between 3 and 12 h. Timing of receipt of a 30 ml/kg fluid bolus over the 12-h period made no difference to mortality despite nearly half the patients having ‘septic shock’ based on the old Sepsis-2 classification. No benefit was seen with timely antibiotics in patients with Gram-positive bacteraemia, and a small benefit was seen with Gram-negative bacteraemia or where no organisms were found. Yet the greatest benefit in terms of risk-adjusted in-hospital mortality (odds ratio 1.15 per hour; 95% CI, 1.07–1.24) was found in ‘other’ bacteraemias that comprised anaerobic, mixed, viral, fungal and yeast cultures. Appropriateness of antibiotics and source control were not evaluated, and confirmation of true sepsis (based on discharge coding) was not performed. Tellingly, benefit was only seen from early bundle completion in the third of the patient population receiving vasopressors; no mortality improvement was seen in the other two thirds. Indeed, another retrospective analysis based on septic patients presenting to the Kaiser Permanente hospital group in Northern California reported a similar effect [19].

Crucially, every prospective study specifically addressing the question of antibiotic delay and outcome fails to show a temporal relationship between early antibiotics and improved survival [20]. What support there is derives from heavily adjusted retrospective analyses of incomplete databases usually collected for other reasons. Even then, the findings are inconsistent [21]. Reasons for delay in delivering antibiotic treatment have also not been ascertained and may be highly pertinent. For example, Filbin et al. recently reported that a third of patients presenting to an emergency department with septic shock had vague symptoms not specific to infection and this represents a previously unrecognized confounder [22]. Notably, a recent paper surveying deaths in six US hospitals found sepsis was present in half the cases and a direct cause of death in a third, yet only 3.7% of the sepsis-associated deaths were adjudged definitely or moderately preventable [23].

There are other examples of a rigid, protocolized approach not yielding outcome dividends. Patients in three prospective multicentre studies [5] assessing the Rivers Early Goal-Directed Therapy strategy [4] failed to show any benefit over standard-of-care. Multicentre trials in African adults [24] and children [25] with sepsis showed worse outcomes when applying a Western-based protocolized approach to management, perhaps related to a logistic inability to deal with iatrogenic complications induced by the approach.

In summary, guidelines are valuable as an aide memoire, but the evidence that strict protocols that demand homogenized management result in outcome benefit is weak at best. Excessive delay is not warranted and, perhaps analogous to managing haemorrhage, the speed of response in an individual septic patient is likely best dictated by their severity [26]. Logically, treatment should be tailored to meet the individual’s needs and underlying comorbidities.

## Repeated trial failures

The last three decades have seen a litany of failed trials of various immunomodulatory and other therapies despite encouraging preclinical data [27]. The methodologies and translatability of many of the underpinning animal models must be challenged as being unfit for purpose [28]. Yet the question must still be posed as to why all have failed to show any outcome benefit when trialled in patients despite a sound scientific rationale and decent proof-of-principle. It is convenient to blame the drug or intervention, but perhaps more focus needs to be placed on the study design itself. To satisfy the great god ‘p’, large numbers of patients need to be recruited into Phase III trials. This drives enrolment of a highly heterogenous population of septic patients in terms of age, underlying comorbidities and source, type and duration of infection, not to mention potential iatrogenic confounders, e.g. variability and/or appropriateness of antibiotics, source control, fluid and vasopressor therapy and ventilator settings. To use but one example, immune function markedly changes with age in healthy subjects [29]. The impact of age has been elegantly demonstrated in preclinical studies where a positive outcome response to antibiotics in young (6–12 weeks old) mice could not be reproduced in an aged (20–24 months) population [30].

Benefit from an intervention in one septic subset may be negated by harm in another, or diluted out by non-effect in a majority subset. This is well illustrated by the SEPSISPAM study [31] where patients were randomized to a high or low mean blood pressure target regardless of their underlying normal blood pressure. No overall mortality difference was noted, yet the likely benefit on renal function of having a higher blood pressure in chronically hypertensive patients was counterbalanced by a harmful signal in normotensives, likely related to injurious side effects of unnecessarily excessive vasopressor doses [32].

It is becoming increasingly apparent that standard physiological and biochemical variables relate poorly to the underlying biological phenotype/endotype/subendotype of the patient. Studies using various ‘omic’ (transcriptomic, metabolomics, proteomic) approaches (e.g. [33–35]) as well as a host of biomarker studies (e.g. [36–38]) demonstrate clear prognostic differences using samples collected even as early as the emergency department. As a useful generalization, eventual non-survivors tend to show a more extreme signature. Other biomarker studies in sepsis and ARDS, albeit using retrospective analyses of trial databases, demonstrate highly variable host and outcome responses to the studied intervention (e.g. fluid, PEEP, corticosteroids, statins) with benefit in some subsets and even harm in others [39–42]. Yet, drug and device trial interventions are generally based on a fixed one-size-fits-all regimen with no consideration given to the patient’s underlying biological phenotype.

As an example, those with a strong pro-inflammatory phenotype may benefit from an appropriate degree of suppression [43]. However, the same dosing regimen may result in excessive suppression in those with a less-pronounced pro-inflammatory signature which can result in an increased propensity to secondary infection/sepsis and, potentially, an increased risk of death. This may explain the variable results seen with corticosteroids which, in recent systematic reviews, appear to give a small degree of survival benefit to patients with septic shock but harm to non-shocked patients [44]. Septic shock patients are more likely to possess a greater pro-inflammatory phenotype [39]. Yet, even within this cohort, the biological signature, and thus the response to the intervention, is still likely to be highly variable.

The advent of rapid point-of-care diagnostics will facilitate appropriate selection of biologically suitable patients into studies, and optimization of drug dosing (when, by how much and for how long). There is no guarantee of success as the derangement in biomarker level may be epiphenomenal rather than integral to a causative pathway, or may even be part of an adaptive process. However, the theranostic targeting of a specific biomarker can be tested a priori for proof-of-principle in appropriate animal models [43].
